# Activation of the *Staphylococcus aureus* intramembrane sensing histidine kinase SaeS via intramembrane interaction with the bacterially encoded small protein ScrA

**DOI:** 10.1128/mbio.01531-25

**Published:** 2025-06-20

**Authors:** Marcus A. Wittekind, Ian R. Monk, Ryan W. Steere, Emily G. Sudnick, Donald Holzschu, Padraig Deighan, Timothy P. Stinear, Ronan K. Carroll

**Affiliations:** 1 Department of Biological Sciences, Ohio University 110002 , Athens, Ohio, USA; 2 Department of Microbiology and Immunology, Doherty Institute for Infection and Immunity, The University of Melbourne 198084, Parkville, Victoria, Australia; 3 Department of Biology, Emmanuel College 10645 , Boston, Massachusetts, USA; 4 Infectious and Tropical Disease Institute, Ohio University 1354, Athens, Ohio, USA; University of Colorado Anschutz Medical Campus, Aurora, Colorado, USA

**Keywords:** two component system, signal transduction, histidine kinase, intramembrane, SaeS

## Abstract

Two-component systems represent a fundamental sense-response mechanism in bacterial cells and often play important roles in critical processes such as quorum sensing, antibiotic tolerance, and virulence. Much has been learned about the role/activity of response regulator proteins, and their impact on gene expression can be monitored using techniques such as RNAseq and ChIP-seq. In contrast, sensor histidine kinases (HKs) remain poorly understood. In most cases, neither the stimulating signal nor the precise mechanism of action is known. This is particularly true for intramembrane sensing HKs (IM-HKs), which sense signals within the bacterial cell membrane. In this study, we show that ScrA, a bacterially encoded transmembrane protein and potent activator of the SaeRS two-component system, interacts directly with the IM-HK SaeS. We demonstrate that the interaction occurs between the transmembrane domains of both proteins and identify three ScrA amino acid residues that are important for this interaction. These results demonstrate that accessory proteins can play an important role in modulating the activity of bacterial two-component systems.

**IMPORTANCE:**

Bacterial pathogens sense environmental stimuli to enable adaptation to new niches, with two-component systems (TCS) playing an important role in this process. TCS consist of a sensor protein that detects a specific signal (often a change in environment), and a response protein that carries out a cellular response (typically altering gene expression). The precise signals that activate TCS are poorly understood, but in general, they are thought to emanate from outside the bacterial cell. Here, we demonstrate that a small membrane protein produced by *Staphylococcus aureus* can play a direct role in the activation of the SaeRS TCS, which plays an essential role in *S. aureus* infection. This represents a novel mechanism of activation for a bacterial TCS.

## OBSERVATION

*Staphylococcus aureus* is a gram-positive opportunistic pathogen, capable of causing life-threatening infections such as endocarditis and bacterial septicemia (1). *S. aureus* can infect a variety of organs/tissues and survive within the host due, in part, to its array of encoded virulence factors, including hemolysins, surface adhesins, and proteases (2–5). Control of such a wide range of virulence factors requires a complex regulatory network made up of sRNAs, alternative sigma factors, standalone protein regulators, and two-component systems (TCS) (6–14). TCS are of particular importance to pathogens like *S. aureus* as they allow the bacteria to sense a variety of signals in the host, including host defense proteins, nutrient/oxygen abundance, and other cells (in the form of quorum sensing) (7, 15–20). Many of the TCS encoded by *S. aureus* are essential for virulence, including the Agr, Arl, and Sae systems (9, 17, 21–27). Interestingly, the regulatory output of some TCS (e.g., the SaeRS system) can be fine-tuned by accessory proteins that alter the activity of the sensor kinase (17, 18, 28–33).

Previous work in our lab identified a small membrane protein ScrA, which stimulates the SaeRS TCS and is required for virulence (34, 35). When overexpressed, ScrA leads to activation of the SaeRS system and spontaneous clumping of *S. aureus* cells due to overproduction of Sae-regulated surface adhesins. While these results clearly indicated that ScrA regulated virulence-related processes through the SaeRS TCS, the molecular mechanism by which ScrA influenced Sae activity was unknown. SaeS, the sensor kinase of the Sae system, is an intramembrane sensing histidine kinase (IM-HK), while ScrA contains one predicted transmembrane helix, leading us to hypothesize that a direct intramembrane interaction between these two proteins leads to activation of SaeS.

### ScrA acts on Sae at a post-transcriptional level

**Fig 1:**
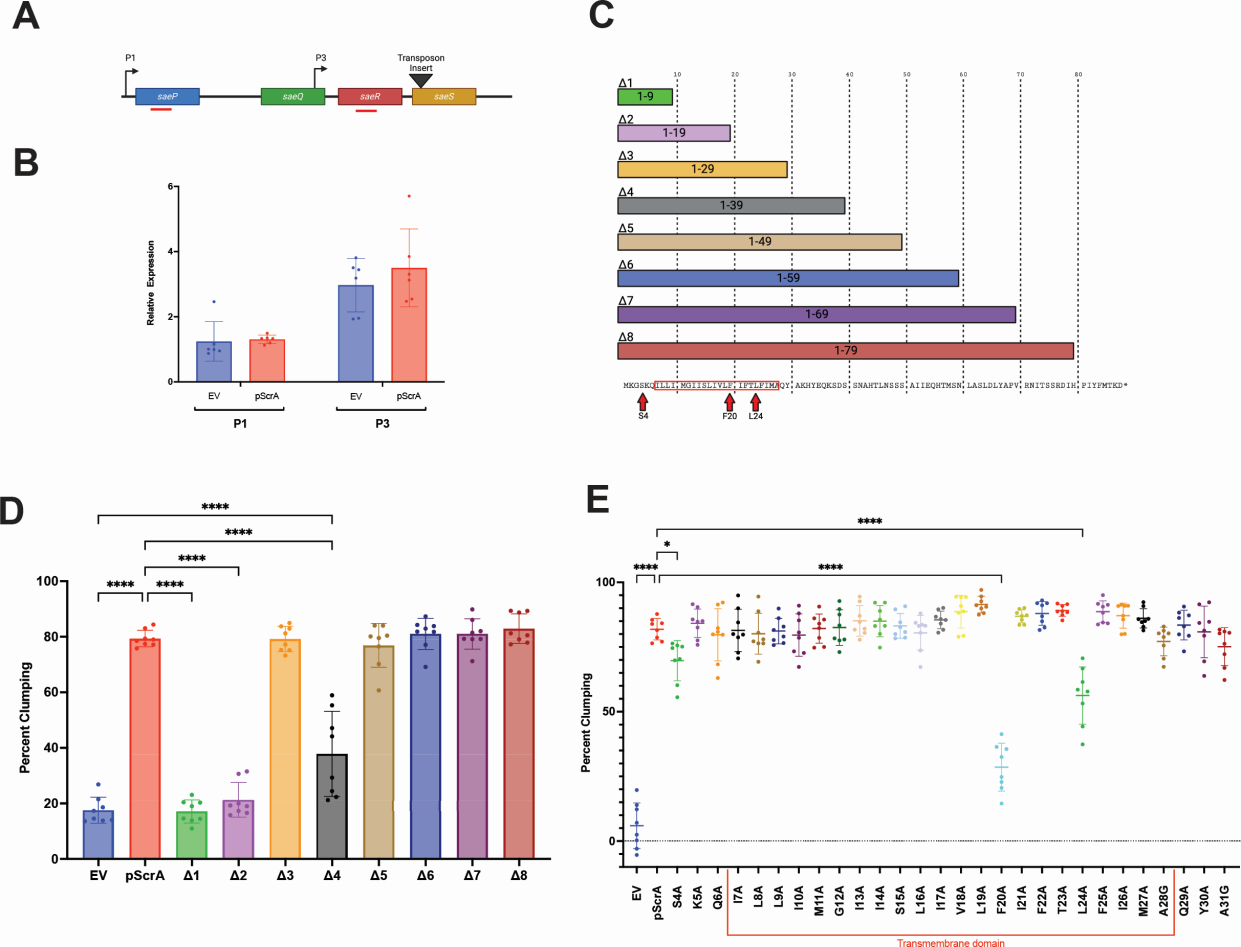
Structure-function analysis of ScrA. (**A**) Schematic of the Sae locus. Location of the *saeS* transposon insertion is indicated by a black triangle, and the approximate location of qPCR products is shown as red bars. (**B**) ScrA was overexpressed from pScrA in an *saeS* mutant background, and the abundance of transcript originating from the P1 and P3 promoters was determined by RT-qPCR. No significant difference in *saeP* or *saeR* transcript was observed in the ScrA overexpressing strains (red bars) compared to empty vector controls (EV, blue bars). Significance was determined by Student’s *t*-test. (**C**) Schematic representation of the eight truncated forms of ScrA, termed Δ1 to Δ8. The ScrA protein sequence is displayed at the bottom. Residues investigated by alanine scanning mutagenesis are depicted by the red box, while alanine substitutions that reduced clumping are marked by red arrows. (**D**) A clumping assay was performed using wild-type *S. aureus* overexpressing each truncated form of ScrA. Decreased clumping was observed in strains expressing Δ1, Δ2, and Δ4. Clumping for strains expressing Δ3 and Δ5–8 was similar to that of strains expressing full-length ScrA protein. Significance was determined by a one-way analysis of variance (ANOVA) using Tukey’s multiple comparison. **** = *P* < 0.001. (**E**) The ScrA transmembrane domain contains three amino acid residues essential for full function. Alanine scanning mutagenesis was performed on 33 amino acids (from positions 4 to 36) within the ScrA transmembrane domain. The resulting proteins were overexpressed in wild-type *S. aureus,* and a clumping assay was performed. Three substitutions (i.e., S4A, F20A, and L24A) significantly reduced clumping activity. Significance was determined by a one-way ANOVA using Tukey’s multiple comparison. * = *P* < 0.5, **** =*P* < 0.001.

Previously, we showed that ScrA overexpression led to activation of the SaeRS two-component system and increased expression of SaeRS-regulated genes. To investigate if ScrA acted at the transcriptional level (i.e., if ScrA activates *saePQRS* transcription), we performed reverse transcriptase-quantitative polymerase chain reaction (RT-qPCR) to determine the abundance of the *saeP* and *saeR* transcripts, which are indicative of Sae P1 and P3 promoter activity, respectively (Fig. 1A). The experiment was carried out in a *saeS* transposon mutant to avoid positive feedback from Sae activation by ScrA, and therefore, the direct impact of ScrA on *sae* transcript abundance was measured. We observed no increase in P1 or P3 transcript levels when overexpressing ScrA (Fig. 1B). This suggests that ScrA is not activating the SaeRS system by increasing transcription of the Sae system genes. Rather, it suggests that ScrA functions at a post-transcriptional level, possibly via a direct protein-protein interaction with SaeS, to influence kinase/phosphatase activity of SaeS.

### The transmembrane domain of ScrA is sufficient for activity

ScrA is an 88 amino acid protein with one predicted transmembrane (TM) domain, from amino acids 7 to 28 (36). Previously, we demonstrated that the predicted TM domain is essential for ScrA-induced cell aggregation (34). To more precisely identify regions of ScrA that influence its activity, we truncated the plasmid-encoded *scrA* gene, generating a total of eight truncations (Fig. 1C). Overexpression of constructs ∆1 and ∆2 (which did not contain the full TM domain) did not lead to an increase in aggregation (Fig. 1D). However, when constructs ∆3 and ∆5–∆8 were expressed, cellular aggregation was observed comparable to that when full-length ScrA protein was overexpressed (Fig. 1D). These results suggest that amino acids after position 29 are dispensable for ScrA function, confirming the TM domain as the region required for ScrA activity. Surprisingly, the expression of construct Δ4 resulted in an intermediate level of clumping. While this result could suggest a repressive role for the amino acids in the 30–39 region, it is also possible that the inclusion of region 30–39 without subsequent residues results in a structural conformation that is less able to interact with target proteins.

### ScrA encodes three amino acids essential for its function

To investigate specific amino acid residues within and/or adjacent to the TM domain that are required for ScrA activity, alanine scanning mutagenesis was performed on the entire TM domain (amino acids 7 to 28), plus three additional amino acids on either side (i.e., from amino acids 4 to 31 of the protein). Substitutions were introduced into a plasmid-encoded copy of the *scrA* gene. As previously observed (34–36), overexpression of wild-type ScrA led to ~80% aggregation (Fig. 1E). Similar results were obtained with most of the alanine-substituted ScrA variants, with three notable exceptions. Substitutions S4A, F20A, and L24A resulted in a significant decrease in cellular aggregation, with the effect being most pronounced with F20A (Fig. 1E). Specifically, reductions of 12%, 53%, and 26% (compared to wild type [WT]) were observed with S4A, F20A, and L24A, respectively. While the levels of clumping were reduced, all three strains still showed an increase in aggregation over the empty vector (Fig. 1E). Equal expression of *scrA* from strains encoding WT ScrA, as well as the S4A, F20A, and L24A substitutions, was confirmed by northern blot (Fig. S1), and peptides corresponding to the C-terminus of ScrA were detected by mass spectrometry in strains expressing the substituted forms. While this non-quantitative analysis does not rule out potential protein stability issues for the substituted forms, it strongly suggests the substituted proteins are being produced. These results demonstrate that three amino acids (S4, F20, and L24), two of which are located within the ScrA TM domain, are important for ScrA-mediated cellular aggregation and therefore are likely important for ScrA structure and/or function.

### ScrA directly interacts with SaeS

**Fig 2:**
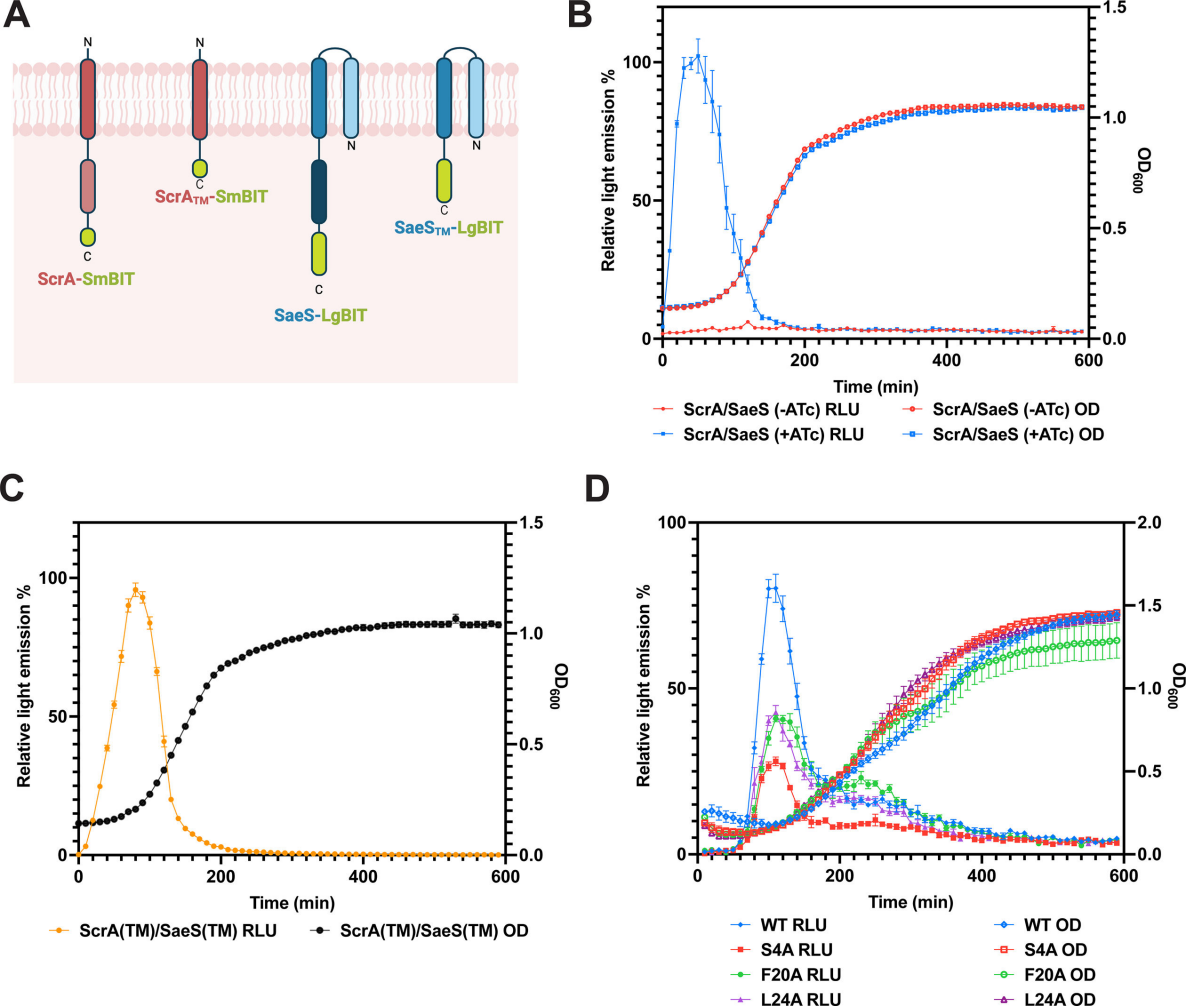
ScrA interacts with SaeS. (**A**) Schematic drawing of the split luciferase fusion proteins used in the analysis. (**B**) Luciferase assay (left hand *y*-axis) and growth curve (right hand *y*-axis) of strains co-expressing ScrA-SmBIT and SaeS-LgBIT. In the absence of anhydrotetracycline induction (red), no light release was detected. Upon induction of both proteins with anhydrotetracycline, an immediate release of light was observed, indicating an interaction between the two fusion proteins. (**C**) Luciferase assay (left hand *y*-axis) and growth curve (right hand *y*-axis) of strain co-expressing the TM domains of ScrA (ScrA_TM_-SmBIT) and SaeS (SaeS_TM_-LgBIT). Production of light indicated an interaction. (**D**) Luciferase assay (left hand *y*-axis) and growth curve (right hand *y*-axis) of strain expressing full-length SaeS and either WT ScrA (blue), ScrA S4A (red), ScrA F20A (green), or ScrA L24A (purple). A reduction in light produced for each of the substituted ScrA proteins indicated reduced interaction between those proteins and SaeS. All experiments were performed at least three times, and representative data are shown. Relative light emission was calculated as a percentage relative to the highest intensity data point. Error bars indicate SEM from triplicate samples.

Given that ScrA and SaeS are transmembrane proteins, and that SaeS is an IM-HK, we hypothesized that activation of the Sae system may occur through a direct interaction between ScrA and SaeS within the *S. aureus* membrane. To test this, we employed a recently developed *S. aureus* split luciferase two-hybrid assay (37) to determine if ScrA directly interacted with SaeS. Plasmid-encoded C-terminal fusion proteins (ScrA-SmBIT and SaeS-LgBIT) were constructed and expressed in WT *S. aureus* cells (Fig. 2A). In the absence of inducer (anhydrotetracycline) or when each plasmid was induced separately within the cell, no light emission was observed. However, when the expression of both fusion proteins was induced within the same cell, a strong increase in light emission (measured as relative light units [RLU]) was observed (Fig. 2B). These results strongly suggest a direct interaction occurs between the ScrA-SmBIT and SaeS-LgBIT proteins. Next, we repeated the assay using fusion proteins containing just the TM domains of ScrA (ScrA_TM_-SmBIT) and SaeS (SaeS_TM_-LgBIT). Once again, a strong increase in light emission was observed, indicating an interaction occurred (Fig. 2C). Collectively, these results strongly suggest an interaction occurs between ScrA and SaeS via their TM domains. Finally, we investigated the contribution of individual ScrA amino acids to the interaction with SaeS. As outlined above, three substitutions (S4A, F20A, and L24A) reduced ScrA-mediated cell aggregation. Due to the location of these residues in/around the TM domain, we hypothesized they may play a role during the interaction with SaeS. Each substitution was introduced into the ScrA-SmBIT fusion protein, and the interaction with SaeS-LgBIT was investigated. Results showed a reduction in light emission for all three substituted forms of ScrA, indicating that each amino acid contributes to the interaction of ScrA with SaeS (Fig. 2D). Importantly, all three substituted forms of ScrA were still observed to interact with SaeS (at a reduced capacity). This is consistent with the data outlined in Fig. 1C whereby each substitution reduced, but did not abolish, activity of ScrA. Experiments performed using a triple substituted form of ScrA containing all three substitutions (S4A, F20A, and L24A) did not show any further reduction in light emission compared to the S4A substituted form alone.

### Conclusion

The biological signals responsible for activating two-component systems are often poorly understood. This is particularly true for IM-HKs. Here, we demonstrate that activation of an IM-HK can occur via interaction of the sensor kinase with a bacterially encoded protein within the bacterial membrane. To our knowledge, this type of “internal” activation is rare. Our previous studies indicated that SrcA plays a particularly important role during infection of the heart (35). It is possible that ScrA exists as a fine-tuning mechanism to activate the SaeS system under specific conditions/environmental niches. Interestingly, we identified two *S*. *aureus* clinical isolates containing amino acid substitutions in ScrA at position F20 (GenBank: HCV1957242.1, HDC3084756.1), further suggesting an important biological role for this amino acid within ScrA. Finally, it is interesting to note that ScrA represents the third *S. aureus*-encoded protein that influences the activity of the Sae system, the others being SaeP and SaeQ.

## SUPPLEMENTAL MATERIAL

Fig. S1 mbio.01531-25-s0001.pdf Northern blot of alanine mutants.

Supplemental text mbio.01531-25-s0002.docx Supplemental materials and methods.

Table S1 mbio.01531-25-s0003.docx Strains used.

Table S2 mbio.01531-25-s0004.docx Oligonucleotides used.

ASM does not own the copyrights to Supplemental Material that may be linked to, or accessed through, an article. The authors have granted ASM a non-exclusive, world-wide license to publish the Supplemental Material files. Please contact the corresponding author directly for reuse.
