# Supplementary material for: Activation of the Staphylococcus aureus intramembrane sensing histidine kinase SaeS via intramembrane interaction with the bacterially encoded small protein ScrA
Source: mBio. 2025 Jun 20;16(7):e01531-25. doi: 10.1128/mbio.01531-25 (PMC12239555; doi:10.1128/mbio.01531-25)
Supplement: Fig. S1 — Northern blot of alanine mutants. [file mbio.01531-25-s0001.pdf]

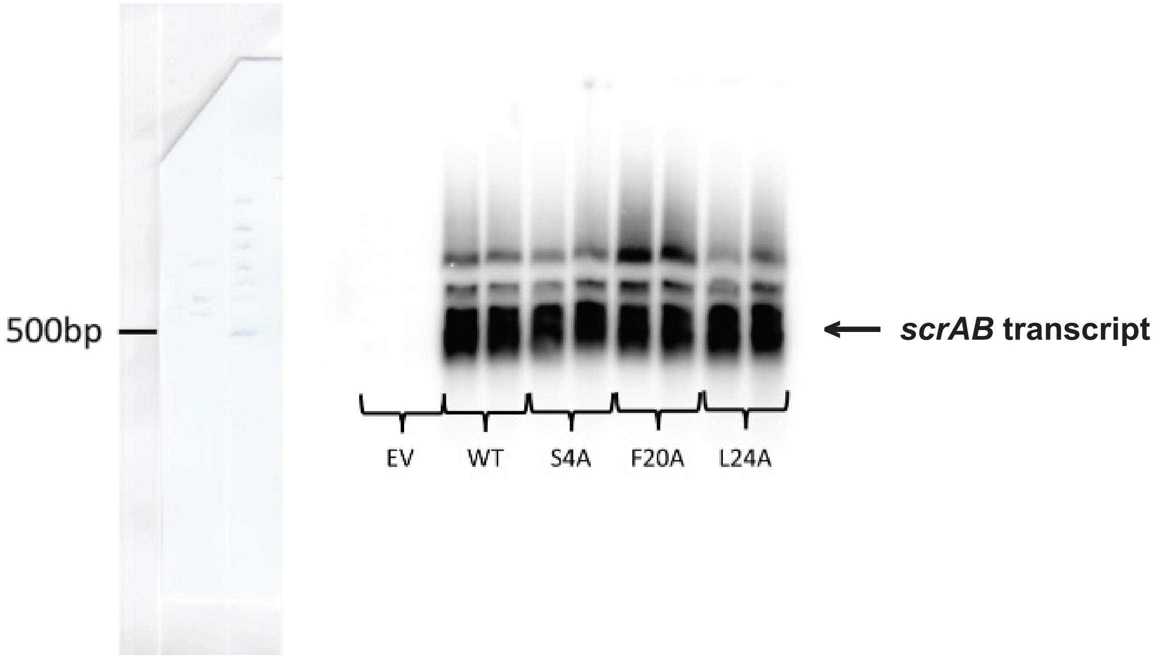

Supplemental Figure S1: Northern blot of alanine mutants.
Alanine scanning mutants showing decrease aggregation were examined by northern blotting to confirm expression of *scrAB* transcript. All overexpression strains showed comparable expression of *scrAB* transcript.
