# Supplementary material for: Activation of the Staphylococcus aureus intramembrane sensing histidine kinase SaeS via intramembrane interaction with the bacterially encoded small protein ScrA
Source: mBio. 2025 Jun 20;16(7):e01531-25. doi: 10.1128/mbio.01531-25 (PMC12239555; doi:10.1128/mbio.01531-25)
Supplement: Supplemental text — Supplemental materials and methods. [file mbio.01531-25-s0002.docx]

**Materials and Methods**

***Bacterial Growth Conditions***

*S. aureus* cultures were routinely grown at 37 °C with shaking in tryptic soy broth (TSB). *E. coli* cultures were grown at 37 °C with shaking in lysogeny broth (LB). Where indicated, antibiotics were used at the following concentrations: Chloramphenicol (10 μg/ml), erythromycin (5 μg/ml), lincomycin (25 μg/ml), ampicillin (100 μg/ml). Where indicated expression was induced with 1 μM CdCl_2_.

***Strains, plasmids, and oligonucleotides***

All bacterial strains and plasmids used in this study are listed in Table S1. All oligonucleotides used are listed in Table S2. The *saeS* transposon mutant was acquired from the Network on Antimicrobial Resistance in *Staphylococcus aureus* (NARSA) (38) and transduced into USA300 AH1263 (39). Phage transduction of both transposon mutation as well as plasmids utilized bacteriophage Φ11. Transformation into AH1263 utilized plasmids purified from *E. coli* strain IM08B (40). Transposon presence was confirmed for *saeS* via PCR utilizing primer pair #1608/#1609.

***Plasmid construction – truncation and alanine scanning***

Construction of alanine scanning mutants and ScrA truncations utilized pRKC1033 (34) as a template with primer pairs as follows. Primers #1273-#1338 were used for ScrA alanine scanning. Primers #1784-#1791 and #1793 were used for ScrA truncations (Table S2). Insertion of fragments and generation of point mutants was performed using an *in vivo assembly* (IVA) method as previously described (41).

***Plasmid construction – two hybrid assay***

PCR amplicons corresponding to *saeR* (IM1401/IM1402), full length ScrA^1-88^ (IM1837/IM1873), and transmembrane ScrA^1-24^(IM1837/IM1839) were amplified from JE2 genomic DNA and cloned by SLiCE into the pSmBIT plasmid, as described previously (37). While the full-length SaeS^1-352^ (IM1487/IM1488) and transmembrane SaeS^1-60^ (IM1487/IM1836) were amplified and cloned into the pLgBIT plasmid. The constructs were sequence validated by ONT sequencing. Combinations of the cloned pSmBIT and pLgBIT plasmids were co-transformed by electroporation with selection on agar plates containing 50 µg/ml kanamycin and 10 µg/ml chloramphenicol. Microtitre plate assays for the kinetic analysis of growth and protein interaction were conducted as described previously (37). When required, 1 ng/ml of ATc was included in the growth media to induce the expression of both tagged proteins. At this concentration, the growth rate of *S. aureus* was not impacted.

***Site directed mutagenesis***

To introduce the S4A, F20A and L24A substitutions into ScrA, the Quickchange site directed mutagenesis kit (Agilent) was used. Primer pairs #2065/2066, #2067/2068, and #2069/2070, were used to introduce the S4A, F20A and L24A substitutions respectively into the pSmBIT_ ScrA^1-88^ plasmid.

***Clumping Assay***

A clumping assay was performed as previously described (34) with minor changes to accommodate more strains. In short, colonies were inoculated into either 5 ml TSB in a 15 ml tube or 600 μl TSB, with appropriate antibiotics and, for pCN51 containing strains, 1 μM CdCl_2_ in a 96 well, deep well plate. Deep well plates were covered with an adhesive seal, while tubes were capped. Both tubes and plates were incubated at 37 °C shaking overnight. For tubes, 1 ml of cells were removed and transferred to a 1.7 ml tube and the top 100 μl of culture were removed and the OD_600_ measured. For 96 well plates cells were resuspended and the top 100 μl of culture were removed to and the OD_600_ determined. Both plates and tubes were incubated statically at room temperature for 2 h. The top 100 μl of culture were removed and the OD_600_ determined. The percent change from the initial OD_600_ was calculated and expressed as the percent clumping.

***RNA Isolations***

RNA was prepared as previously described with minor modifications (34). In short, duplicate overnight cultures were diluted 1:100 and grown for 3 h. Five milliliters of bacterial culture was pelleted and washed with ice‐cold PBS. Pellets were stored at −80°C until use. RNA was isolated using a slightly modified protocol for the RNeasy mini prep kit (Qiagen). RNA samples were treated with a Turbo DNA Free Kit (Ambion). RNA integrity was confirmed via Bioanalyzer (Agilent 2100 Bioanalyzer) and all samples had RIN values >9. RNA was stored at −80°C until use.

***Reverse Transcriptase-Quantitative PCR (RT-qPCR)***

Biological quadruplicates were grown and 1 μg total RNA was reverse transcribed using the iScript cDNA synthesis kit (BioRad) per manufactures instruction. cDNA was diluted 10 times and qPCR was performed using iTaq Universal SYBR Green Supermix (BioRad) in technical duplicates. The housekeeping gene *gyrB* was used as an endogenous control in all reactions. Amplification and analysis were performed as previously described (42).

***Northern Blot***

RNA was isolated from cultures grown for ~16 h as described above. The quantity and purity of the RNA was determined by a bioanalyzer nanochip. 1 μg of RNA was loaded onto a formaldehyde agarose gel and electrophoresed for 1 h 15 min at 120 V. The gel was transferred to a nylon membrane by capillary transfer and RNA was UV crosslinked to the membrane. The ladder and rRNA bands were visualized by staining with a 0.04% methylene blue and 0.5 M sodium acetate solution. To detect the *scrAB* transcript(s) a riboprobe was synthesized as follows: a PCR fragment encompassing the *scrA* open reading frame was synthesized containing a T7 promoter driving antisense expression of *scrA*. This fragment was used as template in an in vitro transcription reaction to generate an antisense riboprobe. The probe was synthesized using α-P^32^ labeled adenosine triphosphate. The membrane was prehybridized for 2 h at 68 °C prior to the addition of the probe. The probe was allowed to hybridize overnight at 68 °C. The membrane was washed with 2X SSC, 1X SSC, and 0.5X SSC, for 15 min each at 68 °C. The membrane was exposed to a phosphor imaging screen overnight and visualized using a phosphor imager.

**REFERENCES**

38. Fey PD, Endres JL, Yajjala VK, Widhelm TJ, Boissy RJ, Bose JL, Bayles KW. 2013. A genetic resource for rapid and comprehensive phenotype screening of nonessential Staphylococcus aureus genes. MBio 4:e00537-12.

39. Boles BR, Thoendel M, Roth AJ, Horswill AR. 2010. Identification of genes involved in polysaccharide-independent Staphylococcus aureus biofilm formation. PLoS One 5:e10146.

40. Monk IR, Tree JJ, Howden BP, Stinear TP, Foster TJ. 2015. Complete Bypass of Restriction Systems for Major Staphylococcus aureus Lineages. mBio 6:e00308-15.

41. Garcia-Nafria J, Watson JF, Greger IH. 2016. IVA cloning: A single-tube universal cloning system exploiting bacterial In Vivo Assembly. Sci Rep 6:27459.

42. Fris ME, Broach WH, Klim SE, Coschigano PW, Carroll RK, Caswell CC, Murphy ER. 2017. Sibling sRNA RyfA1 Influences Shigella dysenteriae Pathogenesis. Genes (Basel) 8.
