# Supplementary material for: Activation of the Staphylococcus aureus intramembrane sensing histidine kinase SaeS via intramembrane interaction with the bacterially encoded small protein ScrA
Source: mBio. 2025 Jun 20;16(7):e01531-25. doi: 10.1128/mbio.01531-25 (PMC12239555; doi:10.1128/mbio.01531-25)
Supplement: Table S1 — Strains used. [file mbio.01531-25-s0003.docx]

**Table S1:** Strains and plasmids used in this study

| Strain or Plasmid | Characteristics | Reference or source |
| --- | --- | --- |
| *S. aureus* |  |  |
| AH1263 | USA 300 LAC isolate cured of plasmid LAC-p03 | (39) |
| NE1296 | JE2 *saeS*::Bursa, NARSA transposon mutant | (38) |
| RKC0630 | AH1263 pCN51 | This Study |
| RKC1039 | AH1263 pRKC1033 (*scrA* overexpression plasmid) | (34) |
| RKC1471 | *saeS*^-^ pCN51 | This Study |
| RKC1472 | *saeS*^-^ pRKC1033 | This Study |
| RKC_1393 | AH1263 pCN51_ScrA_S4A | This Study |
| RKC_1394 | AH1263 pCN51_ScrA_K5A | This Study |
| RKC_1395 | AH1263 pCN51_ScrA_Q6A | This Study |
| RKC_1396 | AH1263 pCN51_ScrA_I7A | This Study |
| RKC_1397 | AH1263 pCN51_ScrA_L8A | This Study |
| RKC_1398 | AH1263 pCN51_ScrA_L9A | This Study |
| RKC_1399 | AH1263 pCN51_ScrA_I10A | This Study |
| RKC_1400 | AH1263 pCN51_ScrA_M11A | This Study |
| RKC_1401 | AH1263 pCN51_ScrA_G12A | This Study |
| RKC_1402 | AH1263 pCN51_ScrA_I13A | This Study |
| RKC_1403 | AH1263 pCN51_ScrA_I14A | This Study |
| RKC_1404 | AH1263 pCN51_ScrA_S15A | This Study |
| RKC_1405 | AH1263 pCN51_ScrA_L16A | This Study |
| RKC_1406 | AH1263 pCN51_ScrA_I17A | This Study |
| RKC_1407 | AH1263 pCN51_ScrA_V18A | This Study |
| RKC_1408 | AH1263 pCN51_ScrA_L19A | This Study |
| RKC_1409 | AH1263 pCN51_ScrA_F20A | This Study |
| RKC_1410 | AH1263 pCN51_ScrA_I21A | This Study |
| RKC_1411 | AH1263 pCN51_ScrA_F22A | This Study |
| RKC_1412 | AH1263 pCN51_ScrA_T23A | This Study |
| RKC_1413 | AH1263 pCN51_ScrA_L24A | This Study |
| RKC_1414 | AH1263 pCN51_ScrA_F25A | This Study |
| RKC_1415 | AH1263 pCN51_ScrA_I26A | This Study |
| RKC_1416 | AH1263 pCN51_ScrA_M27A | This Study |
| RKC_1417 | AH1263 pCN51_ScrA_A28G | This Study |
| RKC_1418 | AH1263 pCN51_ScrA_Q29A | This Study |
| RKC_1419 | AH1263 pCN51_ScrA_Y30A | This Study |
| RKC_1420 | AH1263 pCN51_ScrA_A31G | This Study |
| RKC_1421 | AH1263 pCN51_ScrA_K32A | This Study |
| RKC_1422 | AH1263 pCN51_ScrA_H33A | This Study |
| RKC_1423 | AH1263 pCN51_ScrA_Y34A | This Study |
| RKC_1424 | AH1263 pCN51_ScrA_E35A | This Study |
| RKC_1425 | AH1263 pCN51_ScrA_Q36A | This Study |
| RKC_1434 | AH1263 pCN51_ScrA_Δ1 | This Study |
| RKC_1435 | AH1263 pCN51_ScrA_Δ2 | This Study |
| RKC_1436 | AH1263 pCN51_ScrA_Δ3 | This Study |
| RKC_1437 | AH1263 pCN51_ScrA_Δ4 | This Study |
| RKC_1438 | AH1263 pCN51_ScrA_Δ5 | This Study |
| RKC_1439 | AH1263 pCN51_ScrA_Δ6 | This Study |
| RKC_1440 | AH1263 pCN51_ScrA_Δ7 | This Study |
| RKC_1441 | AH1263 pCN51_ScrA_Δ8 | This Study |
|  |  |  |
| Plasmids |  |  |
| pRKC1033 | pCN51_*scrA* (vector overexpressing *scrA* from a cadmium inducible promoter) | (34) |
| pRKC_1360 | pCN51_ScrA_S4A | This Study |
| pRKC_1361 | pCN51_ScrA_K5A | This Study |
| pRKC_1362 | pCN51_ScrA_Q6A | This Study |
| pRKC_1363 | pCN51_ScrA_I7A | This Study |
| pRKC_1364 | pCN51_ScrA_L8A | This Study |
| pRKC_1365 | pCN51_ScrA_L9A | This Study |
| pRKC_1366 | pCN51_ScrA_I10A | This Study |
| pRKC_1367 | pCN51_ScrA_M11A | This Study |
| pRKC_1368 | pCN51_ScrA_G12A | This Study |
| pRKC_1369 | pCN51_ScrA_I13A | This Study |
| pRKC_1370 | pCN51_ScrA_I14A | This Study |
| pRKC_1371 | pCN51_ScrA_S15A | This Study |
| pRKC_1372 | pCN51_ScrA_L16A | This Study |
| pRKC_1373 | pCN51_ScrA_I17A | This Study |
| pRKC_1374 | pCN51_ScrA_V18A | This Study |
| pRKC_1375 | pCN51_ScrA_L19A | This Study |
| pRKC_1376 | pCN51_ScrA_F20A | This Study |
| pRKC_1377 | pCN51_ScrA_I21A | This Study |
| pRKC_1378 | pCN51_ScrA_F22A | This Study |
| pRKC_1379 | pCN51_ScrA_T23A | This Study |
| pRKC_1380 | pCN51_ScrA_L24A | This Study |
| pRKC_1381 | pCN51_ScrA_F25A | This Study |
| pRKC_1382 | pCN51_ScrA_I26A | This Study |
| pRKC_1383 | pCN51_ScrA_M27A | This Study |
| pRKC_1384 | pCN51_ScrA_A28G | This Study |
| pRKC_1385 | pCN51_ScrA_Q29A | This Study |
| pRKC_1386 | pCN51_ScrA_Y30A | This Study |
| pRKC_1387 | pCN51_ScrA_A31G | This Study |
| pRKC_1388 | pCN51_ScrA_K32A | This Study |
| pRKC_1389 | pCN51_ScrA_H33A | This Study |
| pRKC_1390 | pCN51_ScrA_Y34A | This Study |
| pRKC_1391 | pCN51_ScrA_E35A | This Study |
| pRKC_1392 | pCN51_ScrA_Q36A | This Study |
| pRKC_1426 | pCN51_ScrA_Δ1 | This Study |
| pRKC_1427 | pCN51_ScrA_Δ2 | This Study |
| pRKC_1428 | pCN51_ScrA_Δ3 | This Study |
| pRKC_1429 | pCN51_ScrA_Δ4 | This Study |
| pRKC_1430 | pCN51_ScrA_Δ5 | This Study |
| pRKC_1431 | pCN51_ScrA_Δ6 | This Study |
| pRKC_1432 | pCN51_ScrA_Δ7 | This Study |
| pRKC_1433 | pCN51_ScrA_Δ8 | This Study |
| pRKC_1573 | pSmBIT_ScrA^1-88^ | This Study |
| pRKC_1574 | pSmBIT_ScrA^1-24^ | This Study |
| pRKC_1570 | pLgBIT_SaeS^1-352^ | This Study |
| pRKC_1571 | pLgBIT_SaeS^1-60^ | This Study |
| pRKC_1592 | pSmBIT_ScrA_S4A^1-88^ | This Study |
| pRKC_1583 | pSmBIT_ScrA_F20A^1-88^ | This Study |
| pRKC_1584 | pSmBIT_ScrA_L24A^1-88^ | This Study |
