# Supplementary material for: Activation of the Staphylococcus aureus intramembrane sensing histidine kinase SaeS via intramembrane interaction with the bacterially encoded small protein ScrA
Source: mBio. 2025 Jun 20;16(7):e01531-25. doi: 10.1128/mbio.01531-25 (PMC12239555; doi:10.1128/mbio.01531-25)
Supplement: Table S2 — Oligonucleotides used. [file mbio.01531-25-s0004.docx]

**Table S2:** Oligonucleotides used in this study

| Name | Sequence | Details |
| --- | --- | --- |
| #1608 | CAAAAAAAGAAGCCCTCATTAATGGGAGC | saePQRS locus screen F |
| #1609 | ATTAGTTAATGGCATATTATTTGCCTTCATTTTAAACTTAACTTATCAAATT | saePQRS locus screen R |
| #1824 | ACTGCCAAAACACAAGAACATGA | saeR qPCR F oligo |
| #1825 | CGAGTTCCCTTGGACTAAATGGT | saeR qPCR R oligo |
| #1826 | TCTTCGGCGGCGCTAAATTA | saeP qPCR F oligo |
| #1827 | GGGCTGTGAAATCATACGCTTT | saeP qPCR R oligo |
| #0273 | GGTGCTGGGCAAATACAAGT | gyrB qPCR F oligo |
| #0274 | TCCCACACTAAATGGTGCAA | gyrB qPCR R oligo |
| #1273 | GAAATAATGAAAGGCGCTAAACAAATACTTTTGATTATGGG | pCN51_ScrA S4A IVA Induce Mutation Forward |
| #1274 | GCCTTTCATTATTTCACATC | pCN51_ScrA S4A IVA Induce Mutation Reverse |
| #1275 | ATAATGAAAGGCTCTGCTCAAATACTTTTGATTATGGGCAT | pCN51_ScrA K5A IVA Induce Mutation Forward |
| #1276 | AGAGCCTTTCATTATTTCACAT | pCN51_ScrA K5A IVA Induce Mutation Reverse |
| #1277 | ATGAAAGGCTCTAAAGCTATACTTTTGATTATGGGCATTATATCT | pCN51_ScrA Q61 IVA Induce Mutation Forward |
| #1278 | TTTAGAGCCTTTCATTATTTCACAT | pCN51_ScrA Q6A IVA Induce Mutation Reverse |
| #1279 | AAAGGCTCTAAACAAGCACTTTTGATTATGGGCATTATATCTC | pCN51_ScrA I7A IVA Induce Mutation Forward |
| #1280 | TTGTTTAGAGCCTTTCATTATTTCA | pCN51_ScrA I7A IVA Induce Mutation Reverse |
| #1281 | GGCTCTAAACAAATAGCTTTGATTATGGGCATTATATCTCTTA | pCN51_ScrA L8A IVA Induce Mutation Forward |
| #1282 | TATTTGTTTAGAGCCTTTCATTATTTC | pCN51_ScrA L8A IVA Induce Mutation Reverse |
| #1283 | TCTAAACAAATACTTGCGATTATGGGCATTATATCTCTTATTGT | pCN51_ScrA L9A IVA Induce Mutation Forward |
| #1284 | AAGTATTTGTTTAGAGCCTTTCATTATTT | pCN51_ScrA L9A IVA Induce Mutation Reverse |
| #1285 | AAACAAATACTTTTGGCTATGGGCATTATATCTCTTATTGTTTTATTTA | pCN51_ScrA I10A IVA Induce Mutation Forward |
| #1286 | CAAAAGTATTTGTTTAGAGCCTTT | pCN51_ScrA I10A IVA Induce Mutation Reverse |
| #1287 | CAAATACTTTTGATTGCGGGCATTATATCTCTTATTGTTTTATTTATTTTT | pCN51_ScrA M11A IVA Induce Mutation Forward |
| #1288 | AATCAAAAGTATTTGTTTAGAGCCTT | pCN51_ScrA M11A IVA Induce Mutation Reverse |
| #1289 | ATACTTTTGATTATGGCGATTATATCTCTTATTGTTTTATTTATTTTTACACT | pCN51_ScrA G12A IVA Induce Mutation Forward |
| #1290 | CATAATCAAAAGTATTTGTTTAGAGCC | pCN51_ScrA G12A IVA Induce Mutation Reverse |
| #1291 | CTTTTGATTATGGGCGCTATATCTCTTATTGTTTTATTTATTTTTACACTATTCATC | pCN51_ScrA I13A IVA Induce Mutation Forward |
| #1292 | GCCCATAATCAAAAGTATTTGTTTA | pCN51_ScrA I13A IVA Induce Mutation Reverse |
| #1293 | TTGATTATGGGCATTGCATCTCTTATTGTTTTATTTATTTTTACACTATTCAT | pCN51_ScrA I14A IVA Induce Mutation Forward |
| #1294 | AATGCCCATAATCAAAAGTATTTGT | pCN51_ScrA I14A IVA Induce Mutation Reverse |
| #1295 | ATTATGGGCATTATAGCTCTTATTGTTTTATTTATTTTTACACTATTCATC | pCN51_ScrA S15A IVA Induce Mutation Forward |
| #1296 | TATAATGCCCATAATCAAAAGTATTTGTT | pCN51_ScrA S15A IVA Induce Mutation Reverse |
| #1297 | ATGGGCATTATATCTGCTATTGTTTTATTTATTTTTACACTATTCATCATG | pCN51_ScrA L16A IVA Induce Mutation Forward |
| #1298 | AGATATAATGCCCATAATCAAAAGTATTT | pCN51_ScrA L16A IVA Induce Mutation Reverse |
| #1299 | GGCATTATATCTCTTGCTGTTTTATTTATTTTTACACTATTCATCATGG | pCN51_ScrA I17A IVA Induce Mutation Forward |
| #1300 | AAGAGATATAATGCCCATAATCAAAAG | pCN51_ScrA I17A IVA Induce Mutation Reverse |
| #1301 | ATTATATCTCTTATTGCTTTATTTATTTTTACACTATTCATCATGGC | pCN51_ScrA V18A IVA Induce Mutation Forward |
| #1302 | AATAAGAGATATAATGCCCATAATCAAAA | pCN51_ScrA V18A IVA Induce Mutation Reverse |
| #1303 | ATATCTCTTATTGTTGCTTTTATTTTTACACTATTCATCATGGC | pCN51_ScrA L19A IVA Induce Mutation Forward |
| #1304 | AACAATAAGAGATATAATGCCCATAATC | pCN51_ScrA L19A IVA Induce Mutation Reverse |
| #1305 | TCTCTTATTGTTTTAGCTATTTTTACACTATTCATCATGGC | pCN51_ScrA F20A IVA Induce Mutation Forward |
| #1306 | TAAAACAATAAGAGATATAATGCCCATAATC | pCN51_ScrA F20A IVA Induce Mutation Reverse |
| #1307 | CTTATTGTTTTATTTGCTTTTACACTATTCATCATGGCG | pCN51_ScrA I21A IVA Induce Mutation Forward |
| #1308 | AAATAAAACAATAAGAGATATAATGCCC | pCN51_ScrA I21A IVA Induce Mutation Reverse |
| #1309 | ATTGTTTTATTTATTGCTACACTATTCATCATGGCG | pCN51_ScrA F22A IVA Induce Mutation Forward |
| #1310 | AATAAATAAAACAATAAGAGATATAATGCCC | pCN51_ScrA F22A IVA Induce Mutation Reverse |
| #1311 | GTTTTATTTATTTTTGCACTATTCATCATGGCGCAAT | pCN51_ScrA T23A IVA Induce Mutation Forward |
| #1312 | AAAAATAAATAAAACAATAAGAGATATAATGCC | pCN51_ScrA T23A IVA Induce Mutation Reverse |
| #1313 | TTATTTATTTTTACAGCATTCATCATGGCGCAATAT | pCN51_ScrA L24A IVA Induce Mutation Forward |
| #1314 | TGTAAAAATAAATAAAACAATAAGAGATATAATGCC | pCN51_ScrA L24A IVA Induce Mutation Reverse |
| #1315 | TTTATTTTTACACTAGCCATCATGGCGCAATATGC | pCN51_ScrA F25A IVA Induce Mutation Forward |
| #1316 | TAGTGTAAAAATAAATAAAACAATAAGAGATATAATG | pCN51_ScrA F25A IVA Induce Mutation Reverse |
| #1317 | ATTTTTACACTATTCGCCATGGCGCAATATGCAAAA | pCN51_ScrA I26A IVA Induce Mutation Forward |
| #1318 | GAATAGTGTAAAAATAAATAAAACAATAAGAGATATAATG | pCN51_ScrA I26A IVA Induce Mutation Reverse |
| #1319 | TTTACACTATTCATCGCGGCGCAATATGCAAAACATTAT | pCN51_ScrA M27A IVA Induce Mutation Forward |
| #1320 | GATGAATAGTGTAAAAATAAATAAAACAATAAGAG | pCN51_ScrA M27A IVA Induce Mutation Reverse |
| #1321 | ACACTATTCATCATGGGGCAATATGCAAAACATTATGAACAAAAATC | pCN51_ScrA A28G IVA Induce Mutation Forward |
| #1322 | CATGATGAATAGTGTAAAAATAAATAAAACAATAA | pCN51_ScrA A28G IVA Induce Mutation Reverse |
| #1323 | CTATTCATCATGGCGGCATATGCAAAACATTATGAACAAAAATCC | pCN51_ScrA Q29A IVA Induce Mutation Forward |
| #1324 | CGCCATGATGAATAGTGTAA | pCN51_ScrA Q29A IVA Induce Mutation Reverse |
| #1325 | TTCATCATGGCGCAAGCTGCAAAACATTATGAACAAAAATCC | pCN51_ScrA Y30A IVA Induce Mutation Forward |
| #1326 | TTGCGCCATGATGAATAG | pCN51_ScrA Y30A IVA Induce Mutation Reverse |
| #1327 | ATCATGGCGCAATATGGAAAACATTATGAACAAAAATCCGAC | pCN51_ScrA A31G IVA Induce Mutation Forward |
| #1328 | ATATTGCGCCATGATGAATAG | pCN51_ScrA A31G IVA Induce Mutation Reverse |
| #1329 | ATGGCGCAATATGCAGCACATTATGAACAAAAATCCGACAG | pCN51_ScrA K32A IVA Induce Mutation Forward |
| #1330 | TGCATATTGCGCCATG | pCN51_ScrA K32A IVA Induce Mutation Reverse |
| #1331 | GCGCAATATGCAAAAGCTTATGAACAAAAATCCGACAGTT | pCN51_ScrA H33A IVA Induce Mutation Forward |
| #1332 | TTTTGCATATTGCGCCAT | pCN51_ScrA H33A IVA Induce Mutation Reverse |
| #1333 | CAATATGCAAAACATGCTGAACAAAAATCCGACAGTTC | pCN51_ScrA Y34A IVA Induce Mutation Forward |
| #1334 | ATGTTTTGCATATTGCGC | pCN51_ScrA Y34A IVA Induce Mutation Reverse |
| #1335 | TATGCAAAACATTATGCACAAAAATCCGACAGTTCCA | pCN51_ScrA E35A IVA Induce Mutation Forward |
| #1336 | ATAATGTTTTGCATATTGCGC | pCN51_ScrA E35A IVA Induce Mutation Reverse |
| #1337 | GCAAAACATTATGAAGCAAAATCCGACAGTTCCAAC | pCN51_ScrA Q36A IVA Induce Mutation Forward |
| #1338 | TTCATAATGTTTTGCATATTGCG | pCN51_ScrA Q36A IVA Induce Mutation Reverse |
| #1784 | CAGTATTTATTATGCATTAGAATAGTTACAAAAGTATTTGTTTAGAGCCTTTCATTATTTCACATCCTTTCTAAAAG | pCN51_ScrA Δ1 Reverse |
| #1785 | CAGTATTTATTATGCATTAGAATAGTTATAAAACAATAAGAGATATAATGCCCATAATCAAAAGTATTTGTTTAGAGCCTTTC | pCN51_ScrA Δ2 Reverse |
| #1786 | CAGTATTTATTATGCATTAGAATAGTTATTGCGCCATGATGAATAGTGTAAAAATAAATAAAACAATAAGAGATATAATG | pCN51_ScrA Δ3 Reverse |
| #1787 | CAGTATTTATTATGCATTAGAATAGTTAGTCGGATTTTTGTTCATAATGTTTTGCATATTG | pCN51_ScrA Δ4 Reverse |
| #1788 | CAGTATTTATTATGCATTAGAATAGTTATGATGAATTTAAAGTATGTGCGTTGGAAC | pCN51_ScrA Δ5 Reverse |
| #1789 | CAGTATTTATTATGCATTAGAATAGTTACGACATAGTATGTTGCTCAATTATGGCTGAT | pCN51_ScrA Δ6 Reverse |
| #1790 | CAGTATTTATTATGCATTAGAATAGTTAAGGAGCGTATAAATCTAGAGACGCTAAATTC | pCN51_ScrA Δ7 Reverse |
| #1791 | CAGTATTTATTATGCATTAGAATAGTTAAATATCACGACTAGAAGTAATGTTACGAACAGGAGCGTATAAATC | pCN51_ScrA Δ8 Reverse |
| #1793 | CTATTCTAATGCATAATAAATACTGATAACATCTTATATTTTGTATTATATTTTGTATTATCGTTGACATG | pCN51_ScrA Δ1-9 Forward |
| IM1401 | GATAGAGTATGATGAGGAGGAATTGGAAAATGACCCACTTACTGATCGTGGATG | saeR luc F |
| IM1402 | GAACCACCACCACCACTAGAACCTCGGCTCCTTTCAAATTTATATCCTAATCC | saeR luc R |
| IM1487 | GATAGAGTATGATGAGGAGGAATTGGAAAATGGTGTTATCAATTAGAAGTCAAATC | saeS luc F |
| IM1488 | GAACCACCACCACCACTAGAACCTGACGTAATGTCTAATTTGTGTAATGTTAC | saeS luc R |
| IM1836 | GAACCACCACCACCACTAGAACCAATAAAAATACTACATATTAATAAGGTTAAACAGC | saeS trans luc R |
| IM1837 | GATAGAGTATGATGAGGAGGAATTGGAAAATGAAAGGCTCTAAACAAATACTTTTG | scrA luc F |
| IM1873 | GAACCACCACCACCACTAGAACCATCTTTTGTCATGAAATAAATGGGATG | scrA luc R |
| IM1839 | GAACCACCACCACCACTAGAACCCGCCATGATGAATAGTGTAAAAATAAATAAAAC | scrA trans luc R |
| #2065 | ggaggaattggaaaatgaaaggcgctaaacaaatacttttgattatg | S4A F primer |
| #2066 | cataatcaaaagtatttgtttagcgcctttcattttccaattcctcc | S4A R primer |
| #2067 | gattatgggcattatatctcttattgttttagctatttttacactattcatcatggcgcaatat | F20A F primer |
| #2068 | atattgcgccatgatgaatagtgtaaaaatagctaaaacaataagagatataatgcccataatc | F20A R primer |
| #2069 | ggcattatatctcttattgttttatttatttttacagcattcatcatggcgcaatatgcaaaaca | L24A F primer |
| #2070 | tgttttgcatattgcgccatgatgaatgctgtaaaaataaataaaacaataagagatataatgcc | L24A R primer |
